# ATG7 Limits Basal Antiviral Gene Expression and Moderately Promotes VSV Replication in Mammalian Non-Immune Cells

**DOI:** 10.3390/pathogens15040404

**Published:** 2026-04-08

**Authors:** Xiaohan Tong, Ruixue Wang, Yaxin Liu, Malia B. Potts, Shondra M. Pruett-Miller, Michael A. Whitt, Weikuan Gu, Kui Li

**Affiliations:** 1 Department of Microbiology, Immunology and Biochemistry, University of Tennessee Health Science Center, Memphis, TN 38163, USA; 2 Department of Orthopaedic Surgery and Biomedical Engineering, University of Tennessee Health Science Center, Memphis, TN 38163, USA; 3 Department of Respiratory and Critical Care Medicine, The Second Affiliated Hospital of Harbin Medical University, Harbin 150081, China; 4 Department of Cell and Molecular Biology, St. Jude Children’s Research Hospital, Memphis, TN 38105, USA

**Keywords:** ATG7, vesicular stomatitis virus, autophagy, interferon-stimulated genes, innate immunity, antiviral response

## Abstract

The autophagy regulator ATG7 helps maintain cellular homeostasis and has been suggested to modulate aspects of antiviral immune responses. In Drosophila, ATG7-dependent autophagy contributes to host resistance to vesicular stomatitis virus (VSV), a negative-strand RNA virus of family Rhabdoviridae that is widely used for studying viral biology and developing vaccines and virotherapy. However, the role of ATG7 in mammalian cells, especially non-immune cell types, remains unclear. Herein, we systematically examined the impact of ATG7 on VSV infection using CRISPR-edited cell lines derived from murine embryonic fibroblast (MEF), HeLa, and Huh7.5 cells, in relation to its effect on the expression of antiviral interferon-stimulated genes (ISGs). We found that ATG7 deficiency blocked basal as well as VSV-induced LC3B lipidation, concomitant with moderate reductions in progeny virus yields, while the reconstitution of ATG7 reversed the phenotypes. Mechanistically, ATG7 did not affect viral entry but rather was associated with moderate upregulation of VSV RNA replication. Intriguingly, ATG7 inhibited baseline ISG expression, and this correlated with its pro-VSV effect in all three cell types, while its suppression of innate immune responses elicited post-VSV infection did not. Altogether, these data provide new insights into the role of ATG7 in regulating VSV replication and innate immunity and have implications for developing VSV-based prophylaxis/therapeutics.

## 1. Introduction

Autophagy plays a crucial role in maintaining cellular homeostasis by degrading and recycling damaged or unnecessary cellular components [1]. In recent years, autophagy has garnered increasing attention for its involvement in modulating antiviral immunity, whereby it exhibits complex, sometimes contrary functions that appear to be virus- and/or cell type-specific. On the one hand, autophagy can enhance antiviral defense against some viruses by degrading viral particles and/or promoting immune responses. On the other hand, certain viruses have been shown to hijack the autophagy pathway or specific autophagy-related proteins to facilitate their multiplication and evade host immune surveillance. These opposing roles highlight the intricate relationship between autophagy and viral infections, accentuating the need for a deeper understanding of the regulatory mechanisms that underlie this aspect of host–pathogen interactions [2,3].

Of the ~40 autophagy-related genes (ATGs) that comprise the autophagy machinery, ATG7 is an E1-like enzyme that plays a critical part in the early stages of macroautophagy. In this canonical role, ATG7 catalyzes the activation of ubiquitin-like proteins—ATG8 (including LC3s and GABARAPs) and ATG12—and aids the transfer of these activated proteins to their respective E2 enzymes, ATG3 and ATG10. These processes enable the conjugation of ATG12 to ATG5 and, subsequently, the conjugation of ATG8 to phosphatidylethanolamine (termed ATG8 lipidation), thereby facilitating autophagosomal membrane expansion and, ultimately, autophagosome–lysosome fusion. Interestingly, non-canonical roles of ATG7 have been suggested, including regulating immunity, cell death, and protein secretion, among others [4]. The involvement of ATG7 in viral infections, however, remains poorly understood.

A member of the family Rhabdoviridae with a relatively simple genome and capable of rapid and efficient replication, vesicular stomatitis virus (VSV) is widely used to model the biology of non-segmented negative-strand RNA viruses and as a tool to study viral entry mechanisms when engineered as pseudotypes. Thanks also to its ability to stimulate robust immune responses, its low seroprevalence in human populations, and its preferential replication in and killing of malignant cells, VSV is also a popular vector in vaccine development and oncolytic virotherapy [5,6,7,8,9]. As such, the characterization of host factors that influence cellular permissiveness for VSV and/or immune responses to the virus will aid efforts in furthering the potential of VSV-based medical applications.

In mammalian cells, the interferon (IFN) system plays a central role in guarding against viral infections. This host mechanism is activated upon detecting danger signals—mostly materials deemed “non-self”, such as viral nucleic acids—via innate immune sensor molecules and initiates signaling pathways that drive the expression of IFNs and hundreds of IFN-stimulated genes (ISGs), which collectively are essential for coordinating antiviral immune responses [10,11,12,13]. Due to its high sensitivity to IFNs’ antiviral action, VSV is a long-established tool to gauge IFN antiviral activity in the form of plaque reduction assays and to investigate the effects of cellular and viral factors that regulate IFN immune signaling pathways [14,15,16,17,18,19,20,21]. For its survival advantage, VSV has evolved ways to avoid the efficient expression of IFNs in host cells. One prominent ploy is to shut down host RNA and protein synthesis via its matrix (M) protein, a major virulence factor of VSV [22].

In contrast to vertebrate hosts, insects lack the IFN system and instead rely primarily on RNA interference (RNAi) and, to some extent, autophagy for antiviral defense. In Drosophila melanogaster, autophagy is activated upon VSV challenge, and the loss of ATG7 results in modest increase in viral replication, suggesting that autophagy contributes to controlling VSV infection, although the precise mechanisms remain unclear [23]. Moreover, the autophagy response in Drosophila hemocytes—immune cells in insects—contributes to resistance to several viruses, including VSV [24]. The role of autophagy in the interactions between VSV and mammalian cells is, however, less clear, and published studies have mostly focused on ATG5, which functions as an ATG5–ATG12 conjugate to promote autophagosome formation. It has been shown that, following VSV infection, plasmacytoid dendritic cells (pDCs) employ ATG5-dependent autophagy to deliver VSV RNA from the cytoplasm to endosomes, where viral RNA is detected by toll-like receptor-7 (TLR7) and triggers innate immune activation, leading to the production of IFN-α [17]. By contrast, in murine embryonic fibroblasts (MEFs), ATG5 or the ATG5–ATG12 conjugate dampens the cytoplasmic, retinoic acid-inducible gene I (RIG-I)-dependent IFN antiviral response to VSV infection, resulting in heightened viral growth [16]. Although ATG7 also inhibits the IFN response to synthetic, immune-stimulatory RNA in MEFs [16], how this autophagy-regulatory protein may affect VSV replication and to what extent it operates via its effect on antiviral immune responses remain unclear. This study was conducted to determine the impact of ATG7 on VSV infection, with a focus on mammalian non-immune cells. By interrogating cells of three different tissue origins—fibroblast (MEF), cervix epithelium (HeLa), and liver (Huh7.5) cells with and without genetic deletion of ATG7—we show that ATG7 moderately promotes VSV RNA replication, and this effect cannot be simply interpreted as a result of the ATG7-mediated inhibition of virus-elicited innate antiviral immune responses.

## 2. Materials and Methods

### 2.1. Plasmid Constructs

pCMV-myc-ATG7, encoding the longest isoform (isoform 1) of human ATG7 [25], was a gift from Margrét Ögmundsdóttir (University of Iceland). To construct a retroviral expression vector for untagged human ATG7, we excised the ATG7-coding sequence from pCMV-myc-ATG7 using EcoRI and SacII and inserted it into the multiple cloning sites of pCX4bsr [26]. The resultant construct was designated pCX4bsr-ATG7-iso1. pCX4bsr-TRIM56-FLAG, encoding human TRIM56 fused to a C-terminal FLAG epitope, has been described [27]. The CRISPR/Cas9 construct targeting human ATG7 in the LentiCRISPRv2 puro backbone [28] was provided by Patrick Labonté (INRS-Institut Armand-Frappier, Laval, QC, Canada).

### 2.2. Cell Lines, CRISPR Gene Editing, and Gene Complementation

Mouse embryonic fibroblasts (MEFs), human cervical epithelial carcinoma cell line HeLa, human hepatoma Huh7.5 cells stably reconstituted for expression of TLR3 (Huh7.5-TLR3) [29], human embryonic kidney cell line HEK293, African green monkey kidney cell line Vero, and baby hamster kidney BHK21 cells were used in this study. SV40 T-antigen immortalized MEFs with CRISPR/Cas9-mediated deletion of Atg7 (clone 1C5), referred to as MEF-Atg7KO cells, were provided by the Center for Advanced Genome Engineering (CAGE) at St. Jude Children’s Research Hospital. The gRNA sequence used to create Atg7 KO was 5′-GCACAACACCAACACACUUG-3′. HeLa cells with CRISPR-edited knockout for ATG7, referred to as HeLa-ATG7KO [30], were a gift from L. David Sibley (Washington University in St. Louis, MO, USA). For the reconstitution/complementation of ATG7 expression in MEF-Atg7KO and HeLa-ATG7KO cells, cells were transduced with a replication-incompetent retroviral vector produced in HEK293 cells that were co-transfected with pCX4bsr-ATG7-iso1 and pCL-10A1, using an established procedure [31]. Transduced cells were selected with 5 μg/mL blasticidin for 5 days and then maintained in medium containing 2 μg/mL blasticidin until all untransduced, negative control cells selected in parallel had died. Surviving cell colonies were then pooled and used as ATG7-reconstituted cells, designated MEF-Atg7KO-rcATG7 and HeLa-ATG7KO-rcATG7, respectively. As an irrelevant control for ATG7 expression, MEF-Atg7KO cells were similarly transduced with a retroviral vector carrying TRIM56-FLAG packaged from pCX4bsr-TRIM56-FLAG and stably selected in the presence of blasticidin, yielding the MEF-Atg7KO-T56 cells.

To create Huh7.5-TLR3 cells deficient in ATG7, we conducted CRISPR gene editing using a LentiCRISPRv2 puro-based viral vector specifically targeting human ATG7 [28]. Following selection in puromycin-containing medium that eliminated untransduced cells, limiting dilution in 96-well plates was performed to isolate clonal cell lines, which were subsequently screened for ATG7 deficiency by immunoblotting. Three clonal cell lines completely devoid of ATG7 protein expression were expanded for analyses, along with parental Huh7.5-TLR3 cells, to rule out any effect derived from clonal variation. All cell lines were maintained in Dulbecco’s Modified Eagle Medium (DMEM; Corning, NY, USA) supplemented with 10% fetal bovine serum (FBS; Sigma, St. Louis, MO, USA) and 1% penicillin–streptomycin (CORNING) and cultured at 37 °C in a humidified incubator supplied with 5% CO_2_.

### 2.3. Viruses and 50% Tissue Culture Infectious Dose (TCID_50_) Assay of Virus Infectivity

VSV-Luc is a recombinant VSV expressing the firefly luciferase gene that is inserted between the leader and N gene [32]. VSV-Luc was amplified in Vero cells. VSV-NCP12.1 is a plaque-purified VSV isolate that carries four mutations in the M gene (M33A, M51A, T133A, and S226G) [33]; it also expresses GFP, which is inserted between the viral G and L genes. NCP12.1 is non-cytopathic in BHK21 cells at 24 h post-infection (h.p.i.) but acts with kinetics similar to wild-type (WT) VSV in causing cell rounding in HeLa and HEK293 cells [33]. NCP12.1 was propagated in BHK21 cells. In this study, VSV-NCP12.1 was tested first since this virus would allow a longer observation window post-infection before substantial cytopathic effect (CPE) ensues, at least in some cell types. Subsequent experiments using VSV-Luc, which is of WT VSV backbone, yielded results similar to those obtained using VSV-NCP12.1.

The TCID_50_ assay was performed on Vero cells to measure virus yields in culture supernatant samples collected from infected cells. In short, Vero cells were seeded in 96-well plates to reach ~90% confluence the next day. Supernatant samples were subjected to 10-fold serial dilutions in DMEM supplemented with 2% FBS. One hundred microliters of each dilution were added onto Vero cell monolayers cultured in 100 µL of fresh medium, to reach a final volume of 200 µL per well. Plates were incubated at 37 °C in a humidified atmosphere with 5% CO_2_, and the CPE was examined daily. Final CPE readings were registered at 96 h.p.i. TCID_50_ values were calculated using the Reed–Muench method and expressed as TCID_50_/mL.

### 2.4. VSV Entry Assay

The efficiency of VSV particle entry was assessed using a VSV-G pseudotyped lentiviral vector carrying the firefly luciferase reporter (VSVpp-Luc, a gift from Tong T. Wang, FDA), as described [34]. Briefly, cells were seeded into 96-well plates such that they would reach ~80% confluence the next day. Cells were then infected with VSVpp-Luc in the presence of 8 µg/mL polybrene and incubated at 37 °C for 48 h. Subsequently, the culture medium was removed, and cells were lysed in Passive Lysis Buffer (Promega, Madison, WI, USA). Plates were gently shaken at room temperature for 20 min; then, lysates were centrifuged at 10,000 rpm for 1 min. The supernatant was mixed with firefly luciferase substrate, and luminescence was recorded on a luminometer as per the manufacturer’s instructions (Promega).

### 2.5. Firefly Luciferase Reporter Assay of Viral RNA Replication Using VSV-Luc

VSV-Luc was used to probe viral RNA replication in infected cells. Briefly, cells seeded in 48-well plates were infected by VSV-Luc at an MOI of 0.1 for the indicated times. Cells were then lysed in 50 μL of Passive Lysis Buffer (Promega) and incubated at room temperature for 20 min with gentle shaking. Lysates were centrifuged at 10,000 rpm for 1 min, and the clarified supernatant was subjected to firefly luciferase activity assay (Promega).

### 2.6. RNA Analysis

Total RNA was isolated using TRIzol reagent (Invitrogen, Carlsbad, CA, USA) following the manufacturer’s instructions. Standard reverse transcription (RT) reactions were set up to program the synthesis of cDNA from total RNA using random primers (Invitrogen), followed by quantitative PCR (qPCR) using SYBR Green chemistry and gene-specific primers, as previously described [19,35]. For VSV RNA replication analysis, we used a set of primers in the viral nucleoprotein (N) gene as follows: 5′-CTCTTCTGCTCAGATCCACC-3′ (forward) and 5′-GAGTGTATTTGTTATCTCCAACAC-3′ (reverse). To evaluate the expression of representative antiviral ISGs, qPCR was performed for IFN-induced protein with tetratricopeptide repeats 1 (IFIT1), IFIT2, and IFIT3, or their mouse counterparts, as appropriate. The primers for human IFITs and the 28S housekeeping control have been described [19,20,21,35,36]. The primers for the mouse ISGs were as follows: Ifit1, 5′-AGGCTGGAGTGTGCTGAGAT-3′ (forward) and 5′-AGGGTTTTCTGGCTCCACTT-3′ (reverse); Ifit2, 5′-CGGAAAGCAGAGGAAATCAA-3′ (forward) and 5′-TGAAAGTTGCCATACCGAAG-3′ (reverse); Ifit3, 5′-GCCGTTACAGGGAAATACTGG-3′ (forward) and 5′-CCTCAACATCGGGGCTCT-3′ (reverse) [37]. The relative expression level of each gene was normalized to that of 28S.

### 2.7. Immunoblotting

Cell lysates were prepared and subjected to immunoblotting as previously described [19,20,27,38]. Proteins were resolved by sodium dodecyl sulfate–polyacrylamide gel electrophoresis and transferred onto nitrocellulose membranes using conventional techniques. After incubation in PBS supplemented with 3% nonfat milk to block nonspecific binding sites, membranes were immunoblotted with the following protein-specific monoclonal (mAb) or polyclonal (pAb) primary antibodies: mouse anti-VSV immune ascitic fluid (1:500, ATCC Catalog No. VR-1238AF), rabbit anti-ATG7 pAb (1:1000, Cell Signaling Technology, Danvers, MA, USA), rabbit anti-LC3B mAb (1:1000, Cell Signaling Technology), mouse anti-IFIT3 mAb (1:500, Santa Cruz Biotechnology, Dallas, TX, USA), rabbit anti-ISG56/IFIT1 pAb (1:500) [29], and mouse anti-GAPDH mAb (1:2000, Proteintech, Rosemont, IL, USA). After washing steps, membranes were incubated with IRDye-conjugated secondary antibodies—goat anti-mouse IgG IRDye^®^ 680RD or goat anti-rabbit IgG IRDye^®^ 800CW (LI-COR Biosciences, Lincoln, NE, USA). Signal detection was performed using the Odyssey infrared imaging system (LI-COR Biosciences). Where applicable, protein band intensities were quantified using Image Studio Lite (LI-COR Biosciences), and the expression level for each target was normalized to that of a housekeeping loading control, as specified.

### 2.8. Statistical Analysis

All experiments were performed with multiple independent replicates. Data are expressed as the mean ± standard deviation (SD). Statistical analyses were conducted using the SPSS Statistics 23 software. Statistical significance was assessed using an unpaired Student’s *t*-test or one-way ANOVA as appropriate. A *p*-value of <0.05 was considered statistically significant. Figures were generated using GraphPad Prism 8.

## 3. Results

### 3.1. ATG7 Expression Is Required for Basal as Well as VSV-Induced LC3B Lipidation in MEF and HeLa Cultures

To understand the potential role of ATG7 in VSV infection in mammalian non-immune cell types, we set out to determine the impact of ATG7 deficiency in MEFs and HeLa cells, with the latter being an epithelial cell line derived from human cervical carcinoma. These were selected based on their well-established use in published studies on autophagy, viral replication, and IFN antiviral responses. MEFs, in particular, have been widely used in autophagy research, representing a stable platform for creating knockout models [39]. HeLa cells, known for their efficiency in supporting the replication of many different viruses, serve as a robust model to investigate ATG7 function in human cells [40].

At the outset of this study, we compared MEFs with specific knockout for Atg7 by CRISPR gene editing (referred to as MEF-Atg7KO) and those stably reconstituted/complemented with ATG7 expression via retroviral gene transfer (referred to as MEF-Atg7KO-rcATG7). As an additional control, MEF-Atg7KO was stably transduced with a retroviral vector of the same backbone expressing TRIM56. The resultant cell line, designated MEF-Atg7KO-T56, expresses TRIM56 tagged with a C-terminal FLAG epitope in place of untagged ATG7 in MEF-Atg7KO-rcATG7 cells. TRIM56 was chosen as a control transgene because altering the expression level of this protein per se does not impact VSV replication in mammalian cell cultures [20,27,41,42].

**Figure 1:**
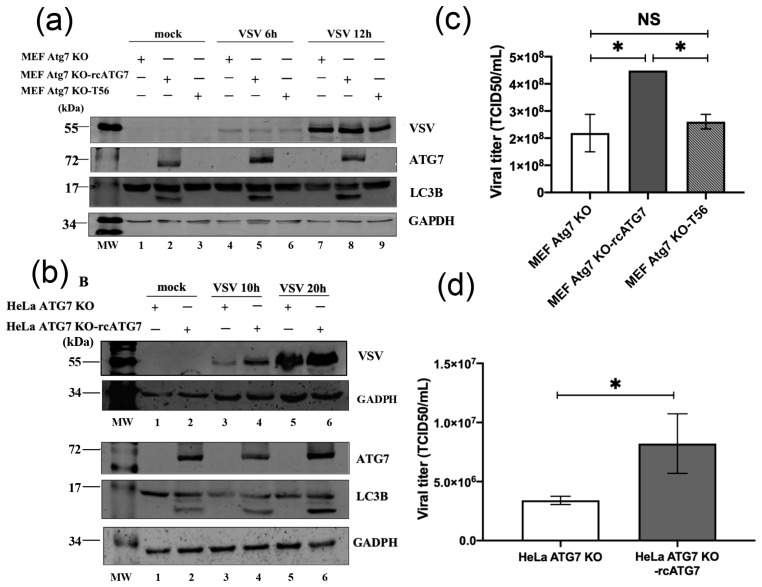
**ATG7 deficiency abrogates basal and VSV-induced LC3B lipidation in MEFs and HeLa cells and significantly reduces VSV yields.** (**a**,**b**) Western blot analysis of VSV, ATG7, LC3B, and GAPDH in the indicated cell lines that were mock-infected or infected by VSV-NCP12.1 (MOI = 0.5) for the indicated times. MW, molecular weight ladder. (**c**,**d**) Progeny virus titers (expressed as TCID50/mL) in culture supernatants of the indicated cell lines at 12 h.p.i. by VSV-NCP12.1. Data represent two independent experiments and are shown as mean ± SD. Asterisks on the lines between bars represent the significance of the differences in viral titers between the indicated groups. * *p* < 0.05. NS, not significant (see Supplementary Materials for the original Western blot of Figure 1).

Immunoblotting revealed the complete absence of ATG7 expression in MEF-Atg7KO cells and, by contrast, readily detectable ATG7 protein in MEF-Atg7KO-rcATG7 cells (Figure 1a, compare lanes 1 and 2). Concomitantly, LC3B was detected exclusively as a single band of the unlipidated form LC3B-I (lane 1) in the former but was expressed as two bands—a predominant LC3B-I and a minor, faster-migrating LC3B-II, i.e., the lipidated form (lane 2). These results confirm that baseline autophagic activity is critically dependent on ATG7 expression in MEFs. Additional evidence supporting the specific role of ATG7 came from MEF-Atg7KO-T56 cells, in which the ectopic expression of TRIM56 neither reversed ATG7 deficiency nor imparted basal LC3B lipidation (Figure 1a, lane 3). Interestingly, we observed a progressive shift from LC3-I to LC3B-II over time following VSV infection in MEF-Atg7KO-rcATG7 cells (Figure 1a, compare lanes 8 and 5 vs. 2), suggesting that autophagic activity is upregulated during VSV infection and that ATG7 facilitates this process by promoting LC3B lipidation. Of note, in the absence of ATG7, LC3B remained solely in the LC3B-I form throughout VSV infection, as observed in MEF-Atg7KO cells (lanes 4 and 7) as well as MEF-Atg7KO-T56 cells (lanes 6 and 9).

Similar results regarding basal and VSV-induced LC3B lipidation were obtained when we examined HeLa cells with genetic deletion of ATG7 (HeLa-ATG7KO) vs. their complemented counterpart in which ATG7 was reconstituted by retroviral gene transfer (HeLa-ATG7KO-rcATG7) (Figure 1b). Taken together, these data establish that ATG7 is required for baseline as well as VSV-induced LC3B lipidation in non-immune cells derived from two mammalian species (mouse and human), regardless of tissue origin (embryonic fibroblast and cervical epithelium). They also suggest that autophagy is heightened during VSV infection and that ATG7 likely plays a critical part in modulating this process in VSV–host cell interactions.

### 3.2. ATG7 Moderately Promotes VSV Propagation in MEF and HeLa Cultures

Having validated the phenotypes of ATG7 knockout and complemented cell lines, we next assessed the impact of ATG7 on VSV propagation by measuring progeny virus production in culture supernatants by the TCID_50_ assay. The results showed that the reconstitution of ATG7 expression significantly, although moderately, promoted VSV replication, with data consistent across different cell types. In MEFs, the viral yield of MEF-Atg7KO-rcATG7 cells was ~2.2-fold higher than that of MEF-Atg7KO cells (*p* < 0.05), suggesting that ATG7 enhances VSV multiplication in this cell type. As a negative control, MEF-Atg7KO-T56 cells gave rise to viral titers comparable to that of MEF-Atg7KO (Figure 1c). The latter observation was in agreement with our previous reports that TRIM56 has no appreciable effect on VSV replication. Moreover, it supported that the pro-VSV effect was ATG7-specific. Likewise, in the HeLa-derived model, the progeny virus titer from HeLa-ATG7KO-rcATG7 cells was ~2.4-fold higher than that from HeLa-ATG7KO cells (*p* < 0.05) (Figure 1d). Collectively, these results illustrate that, while ATG7 is not absolutely required for VSV propagation, there is a consistent, positive correlation between ATG7 expression and VSV multiplication in two mammalian non-immune cell types of different host species.

### 3.3. ATG7 Deficiency Does Not Affect Cellular Entry of VSV

**Figure 2:**
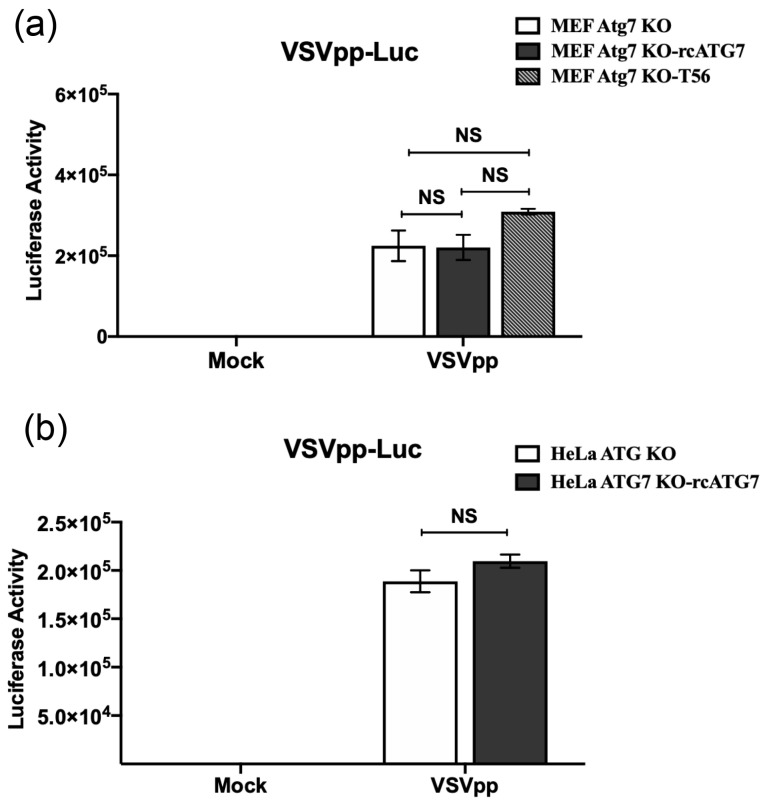
**ATG7 deficiency has no demonstrable impact on cellular entry of VSV.** The indicated cell lines derived from MEFs (**a**) and HeLa (**b**), respectively, were mock-infected or infected by VSVpp expressing firefly luciferase as a readout for efficiency of VSV entry for 48 h, followed by luciferase activity measurement in cell lysates. Data represent two independent experiments and are shown as mean ± SD. NS, not significant.

To understand how ATG7 facilitates VSV multiplication, it was necessary to pinpoint the stage of the viral life cycle at which ATG7 exerts its impact. To this end, we first investigated whether the cellular entry of VSV—an early step in the viral replication cycle—was affected by ATG7 expression status. The VSVpp assay is a widely used platform for assessing factors affecting viral entry, independently of viral replication and subsequent steps [43]. When we exposed ATG7-deficient cells and their complemented counterparts to VSVpp carrying the firefly luciferase reporter gene (VSVpp-Luc), we found no differences in the luciferase activity produced in the cell lysates, in either the MEF- or HeLa-derived models (Figure 2a,b, respectively). These results demonstrate that the presence or absence of ATG7 does not influence the efficiency of VSV entry, suggesting that ATG7 likely modulates a post-entry step in VSV infection to exert its proviral effect.

### 3.4. VSV RNA Replication Takes Place More Efficiently in ATG7-Expressing Cells

**Figure 3:**
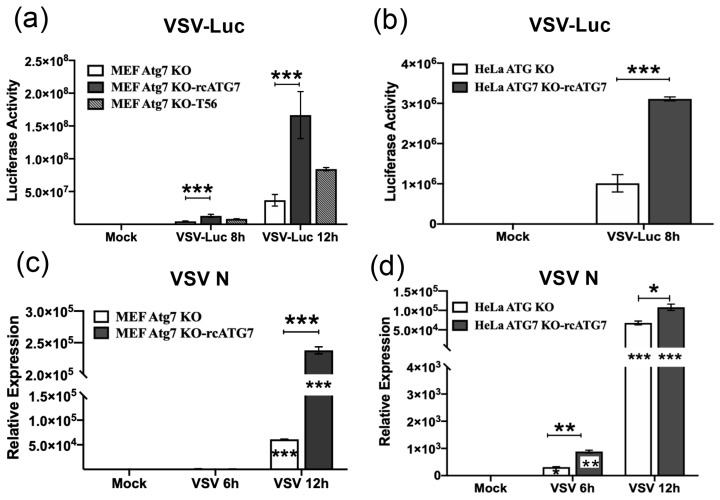
**Complement of ATG7 in MEF-Atg7KO and HeLa-ATG7KO cells positively regulates VSV RNA Replication.** (**a**,**b**) The indicated cell lines were mock-infected or infected by VSV-Luc (MOI = 0.1) for the indicated times, followed by measurement of luciferase activity in cell lysates that served as a readout of VSV RNA replication. (**c**,**d**) qRT-PCR analysis of VSV N RNA levels in the indicated cell lines infected by VSV-NCP12.1 (MOI = 0.5) for the indicated times. Data represent two independent experiments and are shown as mean ± SD. Asterisks inside bars represent the significance of the differences in expression as compared with that cell type’s mock treatment. Asterisks on the lines between bars represent the significance of the differences in expression between the indicated groups. * *p* < 0.05, ** *p* < 0.01, *** *p* < 0.001.

We next assessed the impact of ATG7 on VSV RNA replication, taking advantage of a recombinant VSV encoding the firefly luciferase gene VSV-Luc [32]. A negative-strand RNA virus, the VSV-Luc genome was engineered to contain an antisense strand of the Luc-coding sequence. As such, the expression of the luciferase reporter depends on and is correlated with the replication of the viral RNA in infected cells. By assaying the luciferase activity in cell lysates, the efficiency of VSV-Luc RNA replication can be quantified conveniently and sensitively [18,19,20]. In the MEF-derived model, at 8 h.p.i. of VSV-Luc, the luciferase activity in MEF-Atg7KO-rcATG7 cells was ~2.9-fold higher than in MEF-Atg7KO cells (*p* < 0.001). While the luciferase activity increased robustly in both cultures at 12 h.p.i. as a result of increased viral RNA replication, it remained significantly higher in the former than in the latter, with a ~4.5-fold difference (Figure 3a). These data indicate that ATG7 expression promotes VSV RNA synthesis in MEFs. Similar results were obtained with the HeLa-derived model (Figure 3b). Specifically, we observed that, at 8 h.p.i., the luciferase activity produced in HeLa-ATG7KO-rcATG7 cells was ~3.1-fold higher than in HeLa-ATG7KO cells, confirming the positive regulatory role of ATG7 in VSV RNA replication in another cell type of a different host species.

To corroborate the luciferase reporter-based assay results, we conducted qRT-PCR to measure intracellular VSV N gene RNA levels in cells infected by the VSV-NCP12.1 virus. The results showed that the viral RNA abundance in MEF-Atg7KO-rcATG7 cells was significantly (~3.9-fold) higher than in MEF-Atg7KO cells at 12 h.p.i. (Figure 3c). Likewise, in the HeLa-derived model, we found that HeLa-ATG7KO-rcATG7 cells harbored significantly higher levels of viral RNA than in HeLa-ATG7KO cells at both 6 and 12 h.p.i. (Figure 3d). Notably, data from both assays supported that the presence of ATG7 significantly, albeit moderately, augmented VSV RNA replication, to similar extent as its impact on progeny virus production (Figure 1c,d). Given also our earlier data showing that ATG7 had no appreciable impact on viral entry (Figure 2), we inferred that the primary point whereby ATG7 promotes VSV infection is during the RNA synthesis stage of the viral life cycle. Considering that ATG7 was not absolutely required for VSV propagation (Figure 1), it is likely that ATG7 acts by modulating cellular processes and/or host–virus interactions, creating a cellular microenvironment that is more conducive to viral RNA synthesis.

### 3.5. ATG7 Suppresses Baseline as Well as VSV-Induced ISG Expression in MEF- and HeLa-Based Models

Certain aspects of autophagy and/or autophagy-related proteins are linked to the regulation of innate immune responses, which govern host permissiveness for viral infections. To understand why ATG7 deficiency was associated with moderate reduction in VSV RNA replication, we assessed the expression of three ISGs with anti-VSV activity—IFIT1, IFIT2, and IFIT3 [44,45,46]—at baseline and after VSV infection in ATG7-deficient cells and their complemented counterparts.

**Figure 4:**
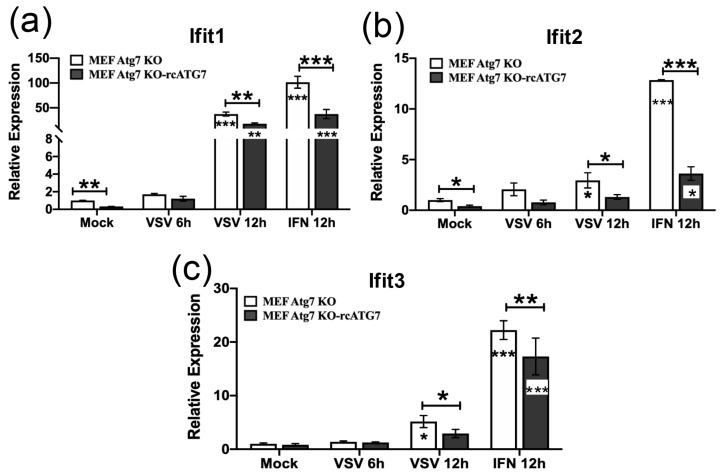
**ATG7 suppresses basal ISG expression, as well as that induced by VSV or IFN-α, in MEFs.** MEF-Atg7KO and MEF-Atg7KO-rcATG7 cells were mock-treated, infected by VSV-NCP12.1 (MOI = 0.5), or incubated with 100 U/mL recombinant mouse IFN-α for the indicated times, followed by total RNA extraction and qRT-PCR analysis of mRNA levels of Ifit1 (**a**), Ifit2 (**b**), and Ifit3 (**c**). Data represent two independent experiments and are shown as mean ± SD. Asterisks inside bars represent the significance of the differences in expression between that treatment and that cell type’s mock treatment. Asterisks on the lines between bars represent the significance of the differences in expression between the indicated groups. * *p* < 0.05, ** *p* < 0.01, *** *p* < 0.001.

In the MEF-based model, qRT-PCR revealed that basal Ifit1 expression was ~3.2-fold higher in MEF-Atg7KO cells than in MEF-Atg7KO-rcATG7 cells. Robust induction of Ifit1 was observed at 12 h.p.i. of the VSV-NCP12.1 virus in both MEF cultures: in MEF-Atg7KO cells, Ifit1 expression was upregulated ~37.3-fold. Although Ifit1 was also considerably induced in MEF-Atg7KO-rcATG7 cells at this time point after infection, its expression level remained significantly (~2.1-fold) lower than in MEF-Atg7KO cells. The same could be said regarding Ifit1 levels after stimulation by murine IFN-α, which served as a positive control for Ifit induction (Figure 4a). We observed a similar trend regarding the expression patterns of Ifit2 (Figure 4b) and Ifit3 (Figure 4c) at baseline and post-stimulation by VSV or murine IFN-α, although the induction of these two ISGs was less pronounced than that of Ifit1, and the difference in the basal expression of Ifit3 between MEF-Atg7KO and MEF-Atg7KO-rcATG7 cells did not reach statistical significance. Together, these data show that the presence of ATG7 negatively regulates the basal as well as VSV-induced expression of Ifits in MEFs.

**Figure 5:**
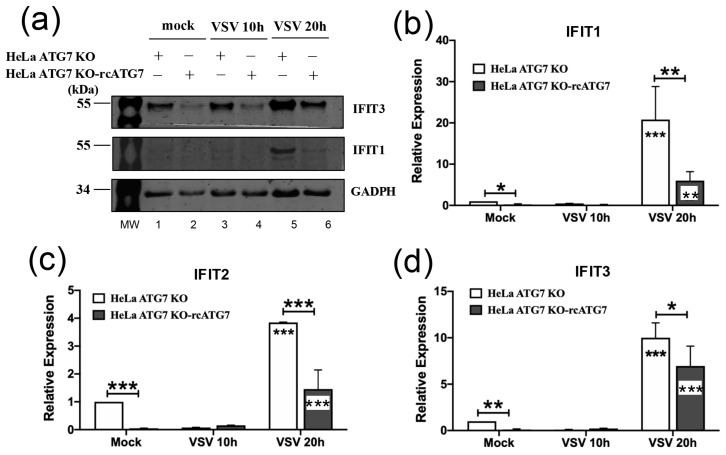
**ATG7 suppresses basal ISG expression, as well as that induced by VSV, in HeLa cells.** HeLa-ATG7KO and HeLa-ATG7KO–rcATG7 cells were mock-infected or infected by VSV-NCP12.1 (MOI = 0.5) for the indicated times, followed by immunoblotting (**a**) and qRT-PCR (**b**–**d**) analyses of the expression of the indicated protein (**a**) and mRNA (**b**–**d**) targets. Data represent two independent experiments. In (**b**–**d**), data are shown as mean ± SD. Asterisks inside bars represent the significance of the differences in expression between that treatment and that cell type’s mock treatment. Asterisks on the lines between bars represent the significance of the differences in expression between the indicated groups. * *p* < 0.05, ** *p* < 0.01, *** *p* < 0.001 (see Supplementary Materials for the original Western blot of Figure 5).

The examination of the HeLa-based model led to similar findings. As shown in Figure 5a, immunoblotting data revealed that the baseline levels of IFIT3 and IFIT1 (compare lanes 1 vs. 2), as well as those induced late post-infection by the VSV-NCP12.1 virus (compare lanes 5 vs. 6), were substantially higher in HeLa-ATG7KO cells than in their counterparts complemented with ATG7. qRT-PCR analysis of the transcript levels confirmed these results and additionally illustrated a similar trend for IFIT2 (Figure 5b–d). In aggregate, data from both the MEF- and HeLa-based cell culture models demonstrate that the baseline and VSV-induced expression of IFITs is suppressed by ATG7 and inversely correlated with cellular permissiveness for VSV.

### 3.6. ATG7 Deficiency Is Associated with Moderate Yet Significant Reduction in Cellular Permissiveness for VSV Replication in Huh7.5-TLR3 Cells, Which Are Defective in RIG-I Signaling

**Figure 6:**
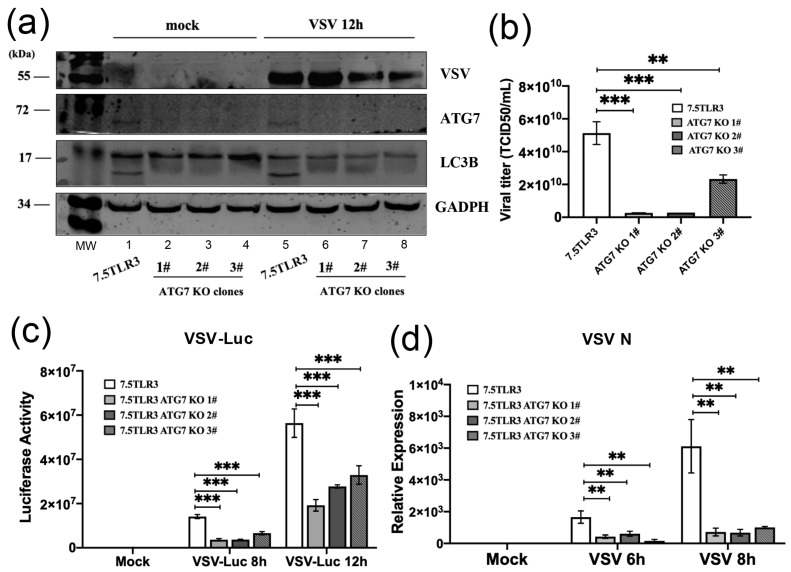
**Knockout of ATG7 impairs VSV replication in Huh7.5-TLR3 cells.** (**a**) WT Huh7.5-TLR3 and Huh7.5-TLR3 ATG7 KO cell lines (clones 1#, 2#, 3#) were mock-infected or infected by VSV-NCP12.1 (MOI = 0.5) for 12 h, followed by immunoblot analysis of VSV, ATG7, LC3B, and GAPDH proteins. (**b**) Progeny viral titers (expressed as TCID50/mL) in culture supernatants of the indicated cell lines at 12 h.p.i. by VSV-NCP12.1 (MOI = 0.5). (**c**) The indicated cell lines were mock-infected or infected by VSV-Luc (MOI = 0.1) for the indicated times, followed by measurement of luciferase activity in cell lysates that served as a readout of VSV RNA replication. (**d**) qRT-PCR analysis of VSV N RNA levels in the indicated cell lines mock-infected or infected by VSV-NCP12.1 (MOI = 0.5) for the indicated times. Data represent two independent experiments. The qRT-PCR data are shown as mean ± SD. Asterisks on the lines between bars represent the significance of the differences between the indicated groups. ** *p* < 0.01, *** *p* < 0.001 (see Supplementary Materials for the original Western blot of Figure 6).

To determine whether the proviral effect of ATG7 on VSV stems from its suppression of basal antiviral gene expression or its inhibition of innate immune responses elicited by viral replication, we conducted experiments in human hepatoma Huh7.5-TLR3 cells with and without ATG7 deficiency. Huh7.5-TLR3 cells, like their parental Huh7.5 line, are defective in RIG-I signaling [29,47] and do not mount an antiviral response to VSV. By CRISPR gene editing of Huh7.5-TLR3 cells, we isolated three independent clonal cell lines with complete loss of ATG7 expression. As shown in Figure 6a, whereas parental Huh7.5-TLR3 cells expressed readily detectable ATG7 protein (lane 1), none of the ATG7 KO cell lines did (lanes 2–4). Mirroring our earlier data obtained from the MEF- and HeLa-based models, baseline LC3B lipidation was abrogated in each of the three Huh7.5-TLR3-ATG7KO lines (compare lanes 2–4 vs. 1). Following challenge by VSV-NCP12.1, a shift from LC3B-I to LC3B-II—indicative of increased autophagy—was observed in parental Huh7.5-TLR3 cells (Figure 6a, compare lanes 5 vs. 1). By contrast, no LC3B-II was detected in any of the three ATG7 KO lines post-infection (lanes 6–8). These data confirm that ATG7 is required for basal as well as VSV-induced LC3B lipidation in this human hepatoma cell model.

The quantification of progeny virus yields in the supernatants of VSV-NCP12.1-infected cultures showed that the viral titers were all significantly lower in the three ATG7 KO cell lines compared to parental (WT) Huh7.5-TLR3 cells (Figure 6b). When we infected cells with VSV-Luc to gauge intracellular viral RNA replication, we observed that the luciferase activity in the cell lysates—proportional to viral RNA replication—at 8 h.p.i. was significantly lower in the three ATG7 KO clones than in the parental Huh7.5-TLR3 lysates, with the differences being 3.8-, 3.8-, and 2.2-fold, respectively. The same trend remained at 12 h.p.i., when VSV RNA replication increased substantially in all four cell lines (Figure 6c). Notably, the VSV-Luc assay results were in line with the VSV-NCP12.1 progeny virus yield data (Figure 6b). Additionally, the viral N RNA abundance in parental Huh7.5-TLR3 cells was significantly higher than in each of the derived ATG7 KO cell lines post-infection, as determined by qRT-PCR (Figure 6d), in line with the VSV-Luc data. These results lend further support to the notion that ATG7 deficiency is associated with moderate yet significant decrease in VSV RNA synthesis in the Huh7.5-TLR3 model, resembling our earlier observations in the MEF and HeLa models.

### 3.7. Greater Basal Expression of IFITs Is Correlated with Reduced VSV Replication in ATG7-Deficient Huh7.5-TLR3 Cells, Which Do Not Mount an Antiviral ISG Response to VSV Infection

**Figure 7:**
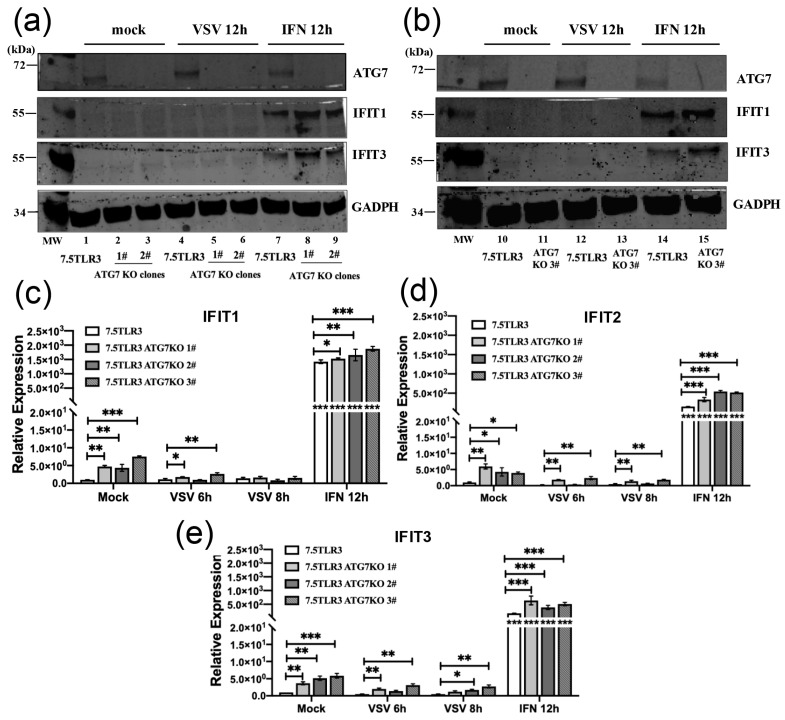
**Basal expression of ISGs is elevated in Huh7.5-TLR3-ATG7KO cells, which do not mount an ISG response to infection by VSV.** (**a**,**b**) WT Huh7.5-TLR3 and three independent Huh7.5-TLR3 ATG7 KO cell lines (clones 1#, 2#, 3#) were mock-treated, infected by VSV-NCP12.1 (MOI = 0.5), or stimulated by incubation with 100 U/mL recombinant human IFN-α for 12 h, followed by Western blot analysis of ATG7, IFIT1, IFIT3, and GAPDH. (**c**–**e**) qRT-PCR analysis of mRNA levels for IFIT1 (**c**), IFIT2 (**d**), and IFIT3 (**e**) in WT Huh7.5-TLR3 and Huh7.5-TLR3 ATG7 KO (clones 1#, 2#, 3#) cells mock-treated or infected by VSV-NCP12.1 (MOI = 0.5) for 6 h and 8 h, respectively, or treated with 100 U/mL IFN-α for 12 h. Data represent two independent experiments. qRT-PCR data are shown as mean ± SD. Asterisks inside bars represent the significance of the differences in expression between that treatment and that cell type’s mock treatment. Asterisks on the lines between bars represent the significance of the differences in expression between the indicated groups. * *p* < 0.05, ** *p* < 0.01, *** *p* < 0.001 (see Supplementary Materials for the original Western blot of Figure 7).

We next determined the expression of IFITs by immunoblotting (Figure 7a) in mock-infected (baseline) and VSV-NCP12.1-infected Huh7.5-TLR3 cells with and without genetic deletion of ATG7. As positive controls for IFIT induction, we set up parallel cultures stimulated by recombinant IFN-α. There was no detectable IFIT1 or IFIT3 protein in uninfected (Figure 7a, lanes 1–3 and 10–11) and VSV-infected cells (lanes 4–6 and 12–13), regardless of ATG7 KO status. This was unlikely a result of inefficient detection, as IFN-α stimulation led to the robust upregulation of IFIT1 and IFIT3 in parental Huh7.5-TLR3 cells as well as in all three Huh7.5-TLR3-ATG7KO lines (lanes 7–9 and 14–15). Of note, moderately heightened expression of IFITs was observed in all three ATG7 KO lines when compared with their WT (parental) counterparts following IFN stimulation (compare lanes 8–9 vs. 7 and lanes 15 vs. 14). To confirm these results and determine if there was any difference in baseline ISG levels, we performed a qRT-PCR, which is more sensitive in detection than immunoblotting, to quantify the expression of the transcript for all three IFITs. There was no upregulation of IFIT1 (Figure 7b), IFIT2 (Figure 7c), or IFIT3 (Figure 7d) in Huh7.5-TLR3 or any of the three ATG7 KO lines post-infection by VSV-NCP12.1, confirming the immunoblotting data (Figure 7a). Since Huh7.5 cells harbor a lethal T55I mutation in the caspase activation and recruitment domain of RIG-I [47], this hepatoma cell line is defective in the RIG-I-mediated IFN antiviral response, including that activated by VSV RNA. Our data herein indicating that the absence of IFIT induction in Huh7.5-TLR3 cells with or without ATG7 KO upon VSV challenge were as anticipated. Nonetheless, they suggest that ATG7’s moderate pro-VSV effect cannot be attributed to the protein’s inhibition of RIG-I-dependent innate immune responses elicited by VSV replication after infection.

qRT-PCR additionally revealed that baseline IFIT1 expression was significantly (~4.4–7.5-fold) higher in all three Huh7.5-TLR3-ATG7KO lines compared with the WT (parental) Huh7.5-TLR3 cells (Figure 7b). The same could be said regarding the basal expression of IFIT2 (Figure 7c) and IFIT3 (Figure 7d), with the ATG7 KO lines consistently having significantly higher expression than their WT counterparts (~3.9–5.9-fold for IFIT2 and ~3.7–5.9-fold for IFIT3, respectively). Consistent with the immunoblotting data (Figure 7a, lanes 8–9 vs. 7 and lanes 15 vs. 14), moderately yet significantly higher expression of IFIT1, IFIT2, and IFIT3 mRNAs was detected in all three Huh7.5-TLR3-ATG7KO lines than in WT cells following IFN-α stimulation (Figure 7b–d). Given that VSV failed to induce an antiviral IFN response (as evidenced by the absence of IFIT induction) in this RIG-I-deficient hepatoma cell model, we conclude that higher basal expression of IFITs (and possibly other antiviral ISGs) likely underlies the moderately reduced replication of VSV in cells with genetic deletion of ATG7.

## 4. Discussion

Using cell lines of three different tissue origins from two host species (murine embryonic fibroblasts, human cervix epithelium, and human hepatocytes), we have demonstrated in this study that ATG7 loss is associated with moderate yet significant reduction in VSV replication in mammalian non-immune cells. The comparison of MEFs and HeLa cells with the genetic deletion of Atg7/ATG7 and their complemented counterparts ascertained that the effect was specific for the ATG7 protein. Adding to the evidence, we have shown that three independent ATG7 KO cell lines derived from human hepatoma Huh7.5 exhibited the same phenotype when compared with their parental WT control cells. The latter data ruled out the small possibility that clonal variation was responsible for the observed proviral effect of ATG7. The VSVpp-based viral entry assay revealed no demonstrable impact of ATG7 deficiency on this initial stage of VSV infection, suggesting that ATG7 affects a post-entry step in the viral life cycle. The quantification of intracellular viral RNA replication using the VSV-Luc assay, as well as qRT-PCR of VSV N RNA levels, supports the notion that the presence of ATG7 facilitates viral RNA synthesis. Given that the extent to which ATG7 loss led to an impairment in VSV RNA replication was in the same range as the degree to which it influenced progeny virus yields, we conclude that intracellular viral RNA replication is the major—if not the sole—point at which ATG7 exerts its pro-VSV action. Thus, in contrast to its reported antiviral role against VSV in insects [23], our data illustrate that an ATG7-dependent mechanism operating in mammalian non-immune cell types is co-opted by VSV for its replication advantage.

In line with the established role of ATG7 as an E1-like enzyme in classical macroautophagy, our data collected from all three mammalian cell types consistently demonstrated that this autophagy-related protein is essential for baseline LC3B lipidation, a key marker of autophagic activity. Additionally, we showed that LC3B lipidation was increased—indicative of heightened autophagy—as VSV infection progressed and that this process was also critically dependent on ATG7. These observations hint that the classical autophagy pathway is activated late post-infection by VSV in mammalian cells, as was the case in Drosophila [23], and likely has a role in regulating the viral replication cycle. Considering our data showing that ATG7 KO only moderately compromised VSV RNA replication, it is plausible to reason that ATG7-mediated LC3B lipidation is not absolutely required for viral RNA synthesis but rather orchestrates cellular processes that impact the microenvironment in ways conducive to VSV multiplying its genetic material. Given that autophagosomes can still be formed in response to certain stressors via an ATG5/ATG7-independent alternative macroautophagy not involving LC3B lipidation [48], whether and to what degree the pro-VSV effect observed herein stems from ATG7-dependent autophagy or an autophagy-independent role of this autophagy-related protein will require further investigation.

Apart from its ancient, homeostatic role, the autophagy pathway has been suggested to modulate host immunity against microbial infections. Dependent on the cell type and cellular context, both positive and negative effects of autophagy on host innate immune responses to viruses have been reported. Specifically for ATG7, limited information suggests that this protein negatively influences the type I IFN response to stimulation by cytosolic poly-I:C, a double-stranded (ds) RNA surrogate, which engages the cytoplasmic viral RNA sensors RIG-I and MDA5, with Atg7 KO MEFs producing higher amounts of IFN-α and IFN-β than WT MEFs [16]. Our data comparing ATG7-deficient and -complemented MEFs and HeLa cells confirmed that ATG7 limits the VSV-induced upregulation of antiviral IFITs, which is known to be mediated by RIG-I. Interestingly, our study additionally found that the presence of ATG7 constrained the baseline expression of the IFITs in uninfected cells, suggesting that it limits antiviral responses to endogenous ligands to maintain cellular homeostasis and avoid deleterious autoimmune activation. The precise nature of the endogenous ligands and the pattern recognition receptor that they engage with are not known. A possible candidate is the inverted *Alu* repeat—a duplex RNA element that activates MDA5, especially during insufficiency of ADAR1, a dsRNA-editing enzyme [49]. We arrive at this hypothesis based on our data indicating that basal ISG expression was also suppressed by ATG7 in Huh7.5-TLR3 cells, which were RIG-I-deficient. At present, we favor the interpretation that the ATG7-mediated inhibition of basal antiviral gene expression is unfortunately exploited by VSV for its replication advantage, while the ATG7-dependent curtailment of virus-elicited antiviral ISG responses post-infection (presumably via autophagy) is not as critical. Several lines of evidence support this argument. First, the extent of ATG7’s proviral effect was comparable among MEF-, HeLa-, and Huh7.5-TLR3-based models, even when no ISG response to VSV infection was detectable in Huh7.5-TLR3 cells because of an RIG-I-defective mutation. Despite the considerable induction of antiviral IFITs post-VSV infection being observed in HeLa cells and MEFs, ATG7 did not have a greater impact on VSV replication than in Huh7.5-TLR3 cells. Second, the basal expression of IFITs was inversely correlated with cellular permissiveness for VSV. Specifically, IFIT proteins were readily detectable in the HeLa-based model in uninfected cells and more so in HeLa-ATG7-KO cells, whereas no IFIT proteins could be detected in Huh7.5-TLR3 cells before and after VSV infection, regardless of ATG7 KO. Concordant with their higher basal levels of IFITs, HeLa cells were three orders of magnitude less permissive than Huh7.5-TLR3 cells for producing infectious VSV particles (compare Figure 1d vs. Figure 6b). It should be noted, however, that while our study demonstrates that ATG7 has a moderate promoting effect on VSV replication in both mouse and human non-immune cells, which is accompanied by a reduction in baseline ISG expression, whether there is a causal relationship between the two effects remains to be investigated. As the evidence stands, we can only suggest that these two are correlated. The precise mechanism by which ATG7 exerts its proviral role in VSV replication warrants further study, and it may involve more complex interactions among the virus, autophagy, and innate RNA-sensing mechanisms.

One interesting question to be addressed in future studies is whether the ability of ATG7 to promote VSV replication is mediated through its suppression of the IFIT family of antiviral proteins. Toward this end, experiments in which the expression of IFIT1, IFIT2, and IFIT3 is depleted by RNAi or genetic ablation in cells with and without ATG7 KO will inform about whether ATG7 retains its proviral effect in the absence of these known anti-VSV effectors. However, one caveat is that other antiviral proteins that are basally expressed in uninfected cells may also contribute, to some extent, to the ATG7 regulation of cellular permissiveness for VSV.

In summary, this study delineates a proviral role of ATG7 in the context of VSV infection of mouse and human non-immune cell types that involves moderately more efficient viral RNA synthesis in ATG7-competent cells, pointing to an intriguing link between ATG7-mediated suppression of basal antiviral ISG expression in host cells and enhanced VSV multiplication. These new revelations add to the knowledge of ATG7 in host–VSV interactions and the regulation of innate antiviral immunity and may have implications for the refinement of strategies for developing VSV-based therapeutic and/or preventive applications.

## Data Availability

All data are contained within the article. Raw data can be made available upon request.

## Supplementary Materials

The following supporting information can be downloaded at: https://www.mdpi.com/article/10.3390/pathogens15040404/s1, File S1: The original Western blot images for Figure 1, Figure 5, Figure 6 and Figure 7.
