# Supplementary material for: ATG7 Limits Basal Antiviral Gene Expression and Moderately Promotes VSV Replication in Mammalian Non-Immune Cells
Source: Pathogens. 2026 Apr 8;15(4):404. doi: 10.3390/pathogens15040404 (PMC13118527; doi:10.3390/pathogens15040404)
Supplement: Supplementary file 1 [file pathogens-15-00404-s001.zip › pathogens-4249990 - Western Blot Original Pictures.pptx]

## Slide 1
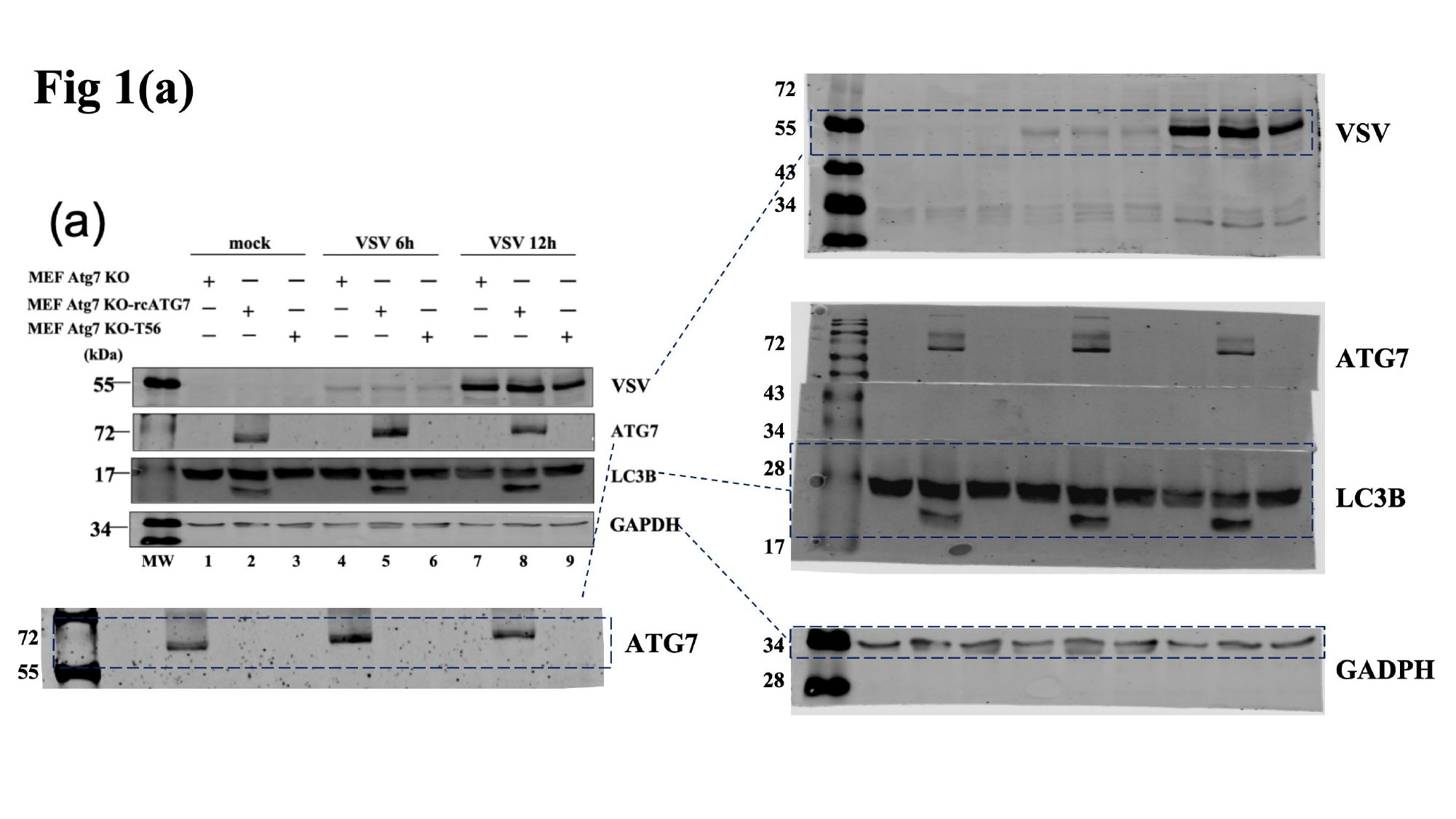

Fig 1(a)
72
55
43
34
72
43
34
28
17
34
28
VSV
ATG7
LC3B
GADPH

## Slide 2
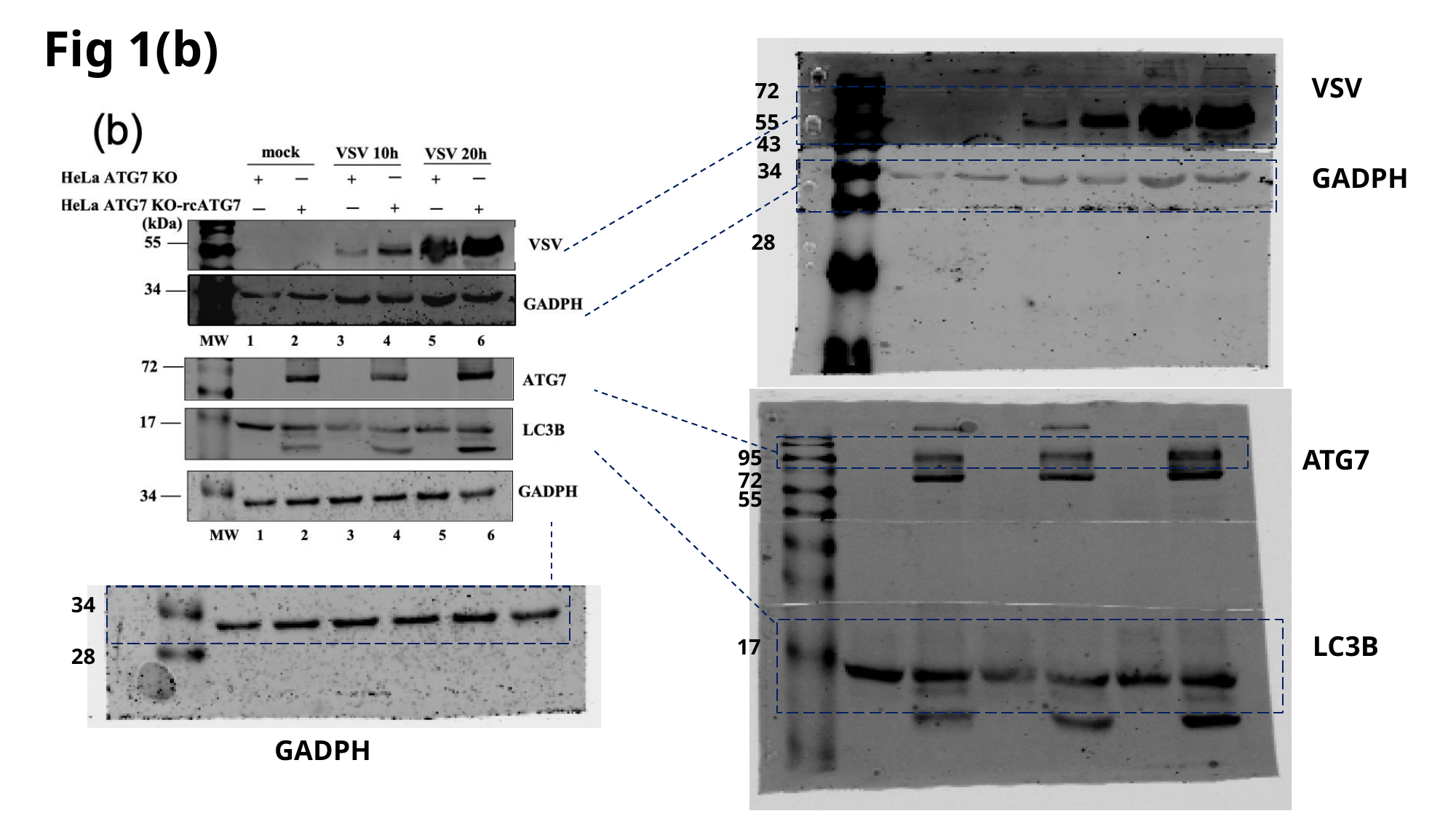

Fig 1(b)
VSV
72
55
43
34
GADPH
28
95
72
55
17
ATG7
34
28
GADPH
LC3B

## Slide 3
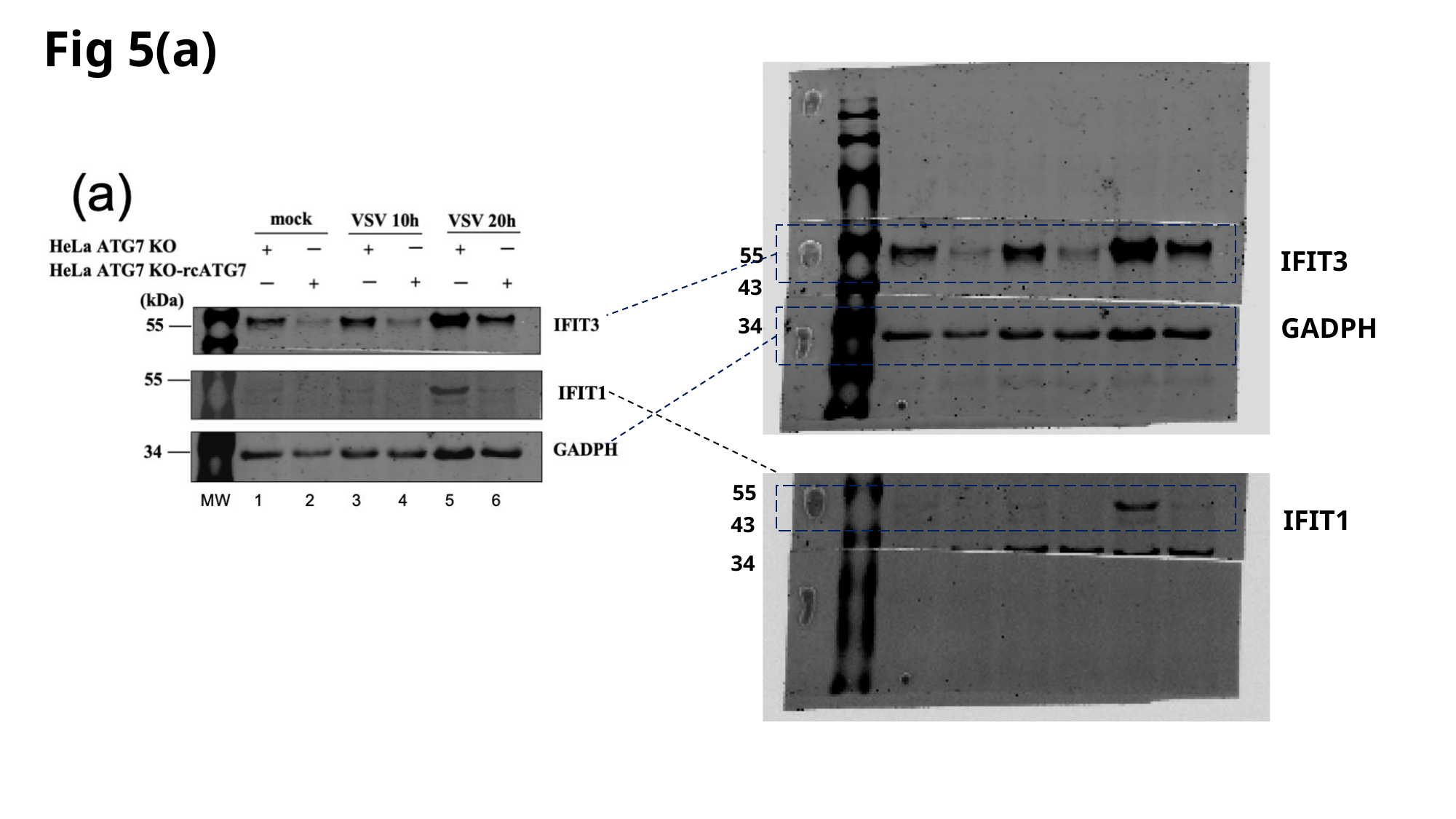

Fig 5(a)
55
IFIT3
43
GADPH
34
55
IFIT1
43
34

## Slide 4
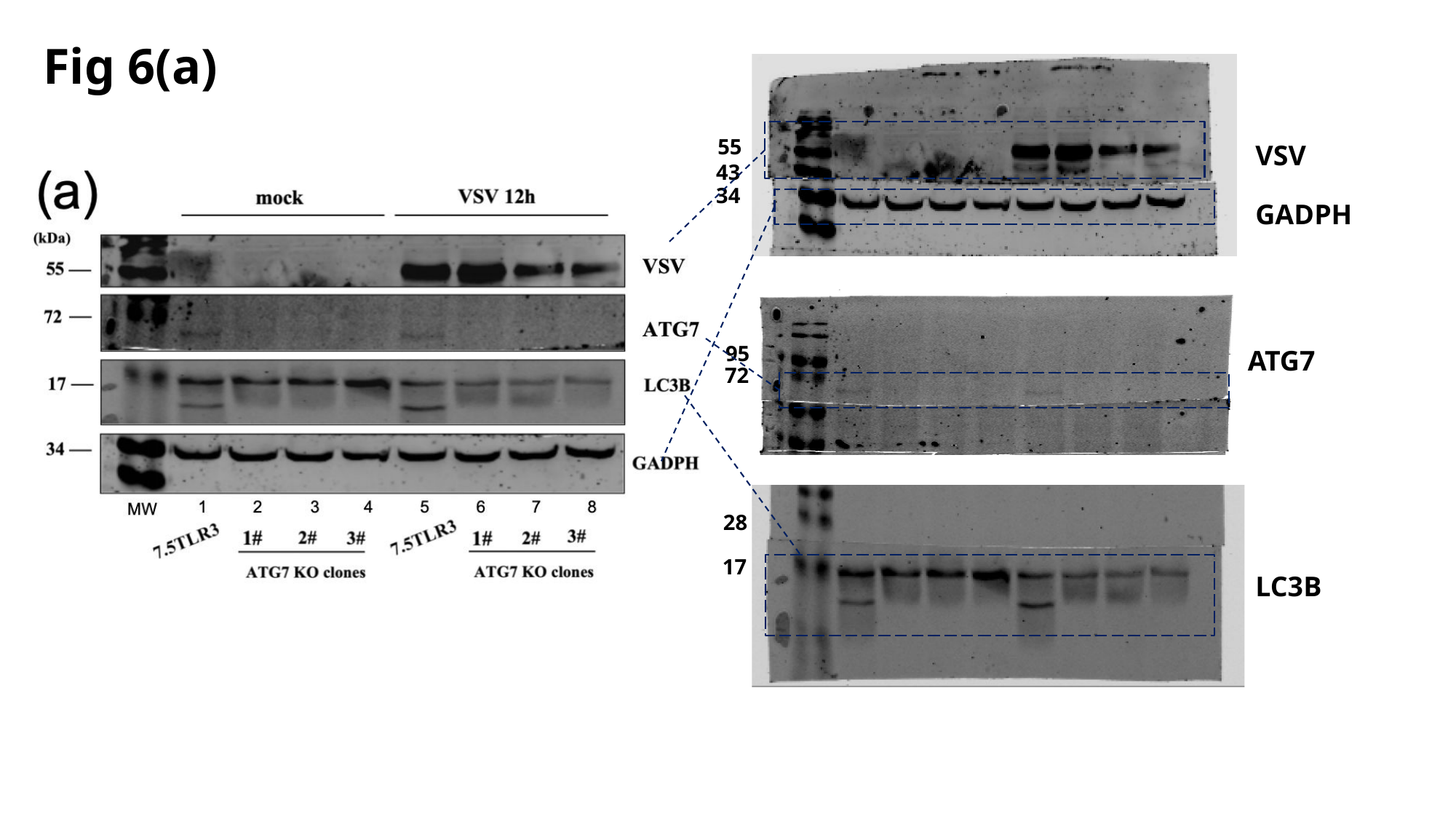

Fig 6(a)
55
43
34
95
72
28
17
VSV
GADPH
ATG7
LC3B

## Slide 5
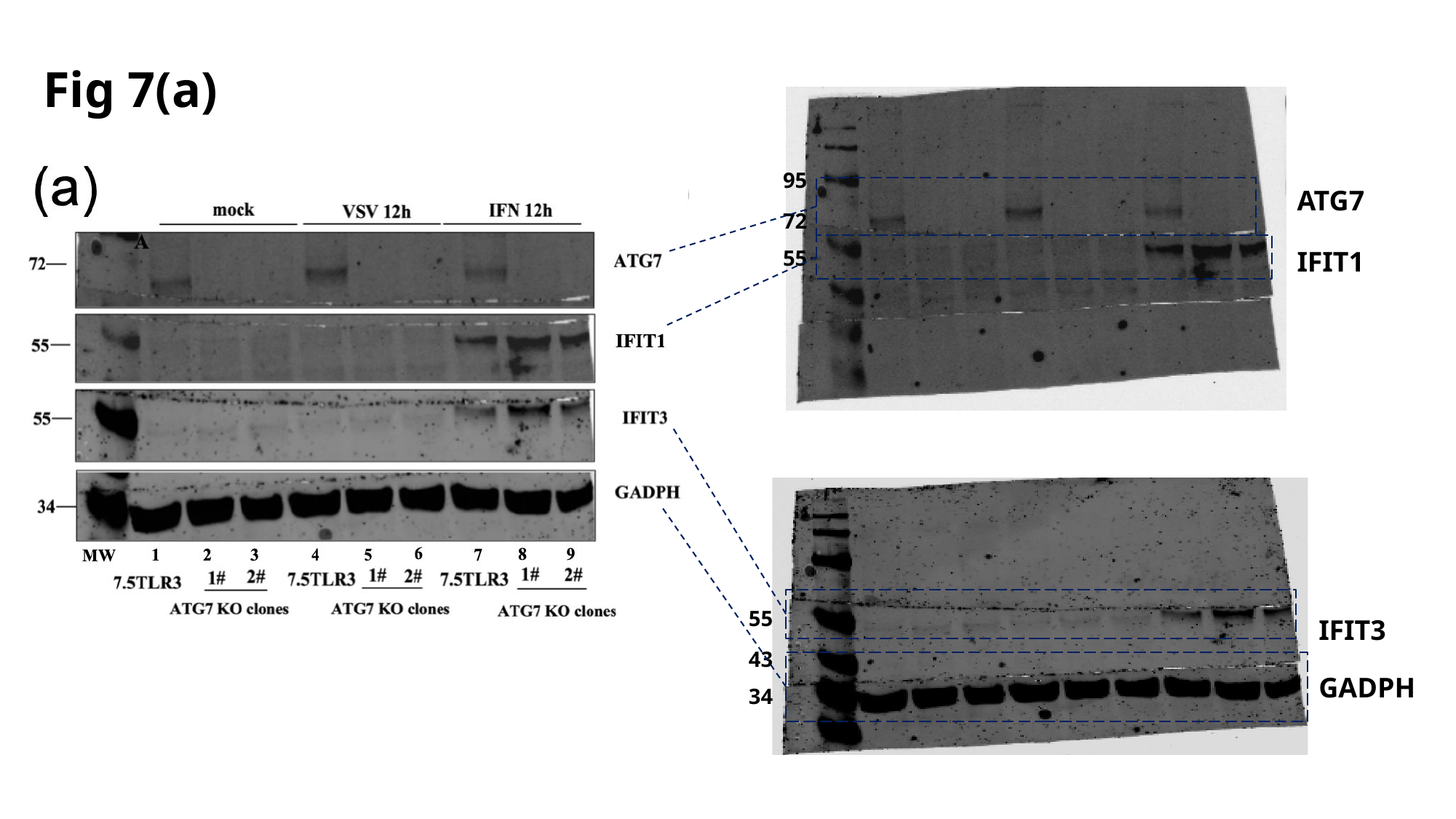

Fig 7(a)
95
ATG7
72
55
IFIT1
55
43
34
IFIT3
GADPH

## Slide 6
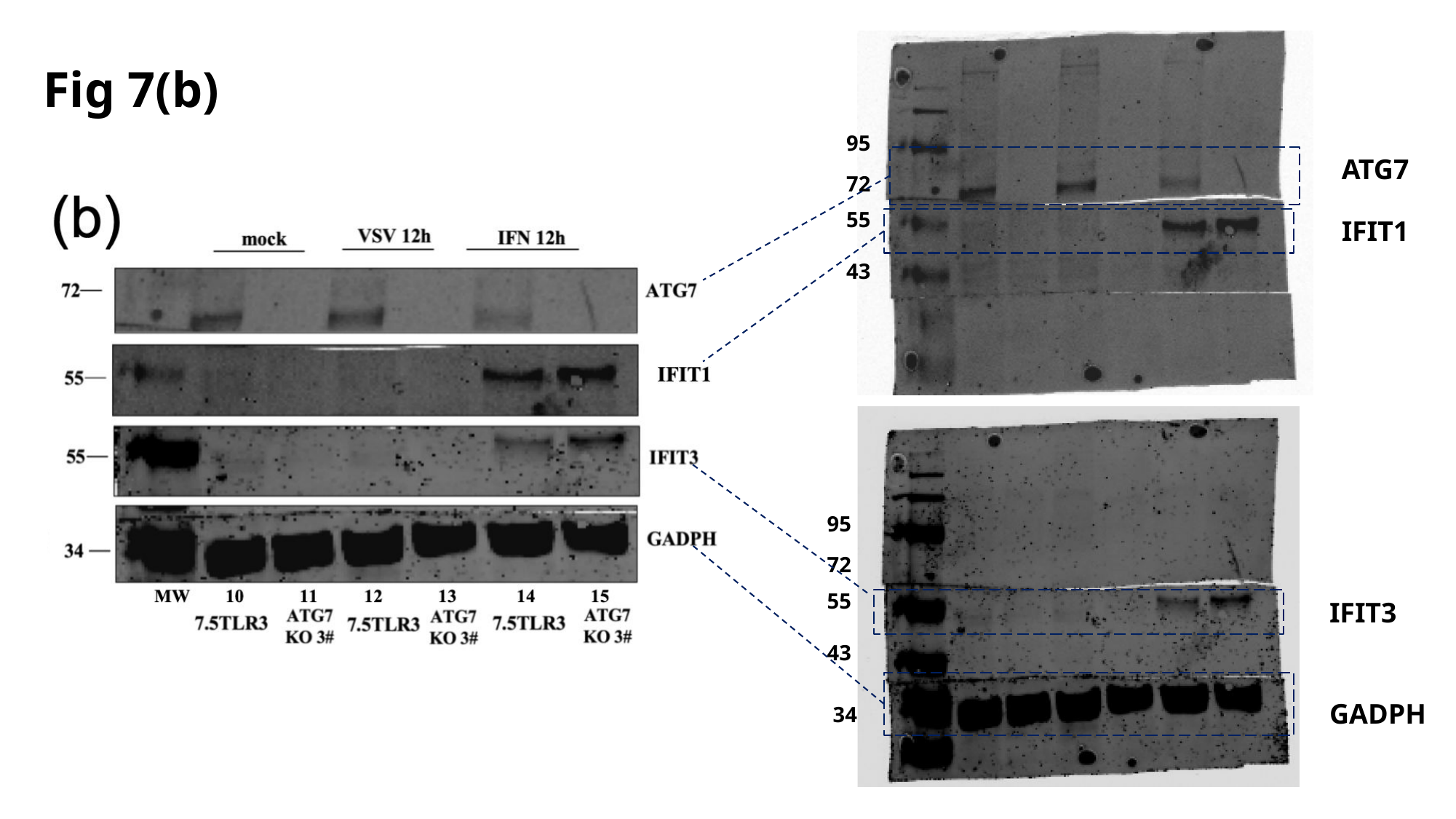

Fig 7(b)
95
ATG7
72
55
IFIT1
43
95
72
55
IFIT3
43
GADPH
34
