# Supplementary figures and images for: ATG7 Limits Basal Antiviral Gene Expression and Moderately Promotes VSV Replication in Mammalian Non-Immune Cells
Source: Pathogens. 2026 Apr 8;15(4):404. doi: 10.3390/pathogens15040404 (PMC13118527; doi:10.3390/pathogens15040404)

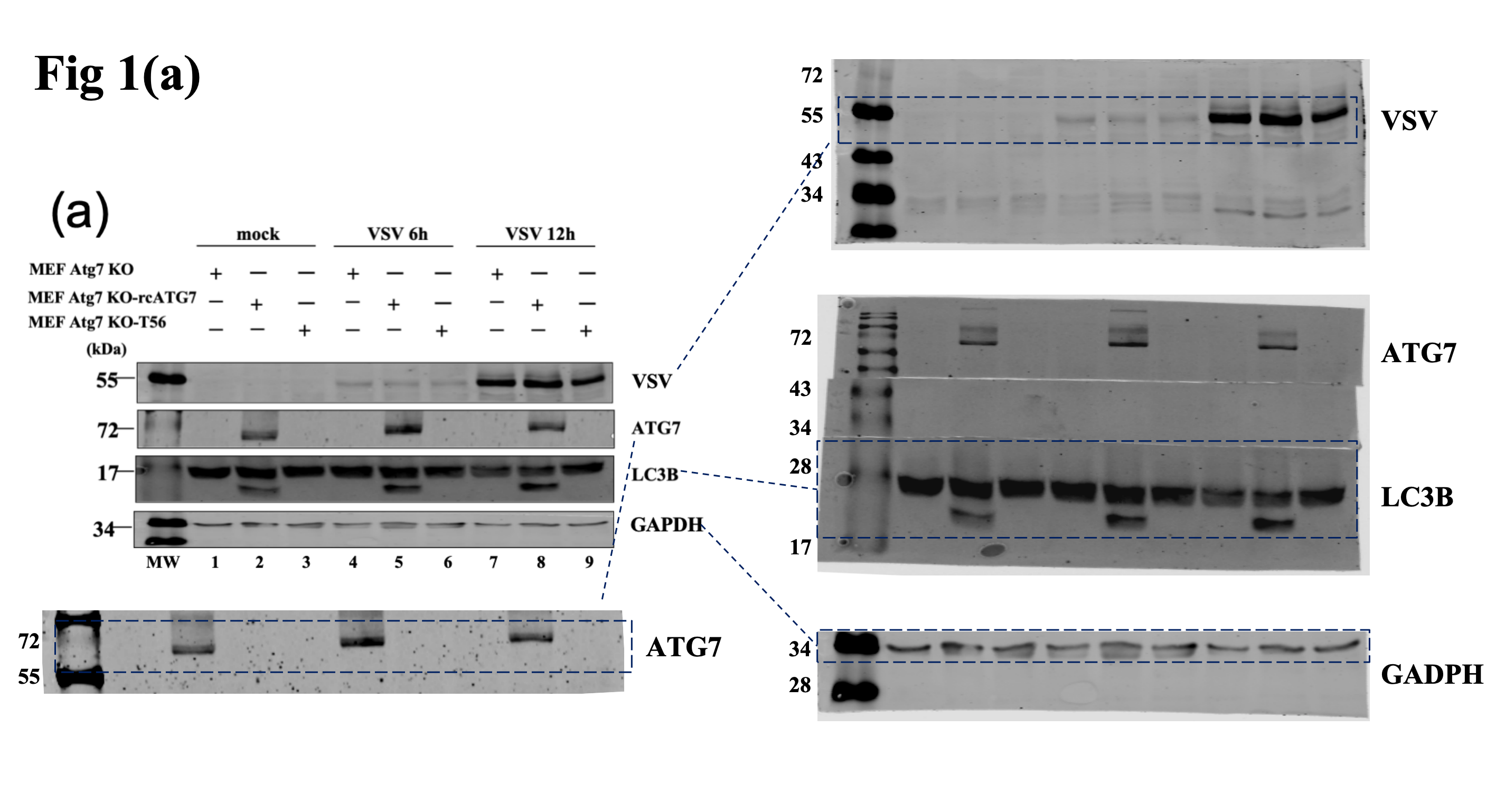

Supplement: Supplementary file 1 [file pathogens-15-00404-s001.zip › Fig1(a).tif]

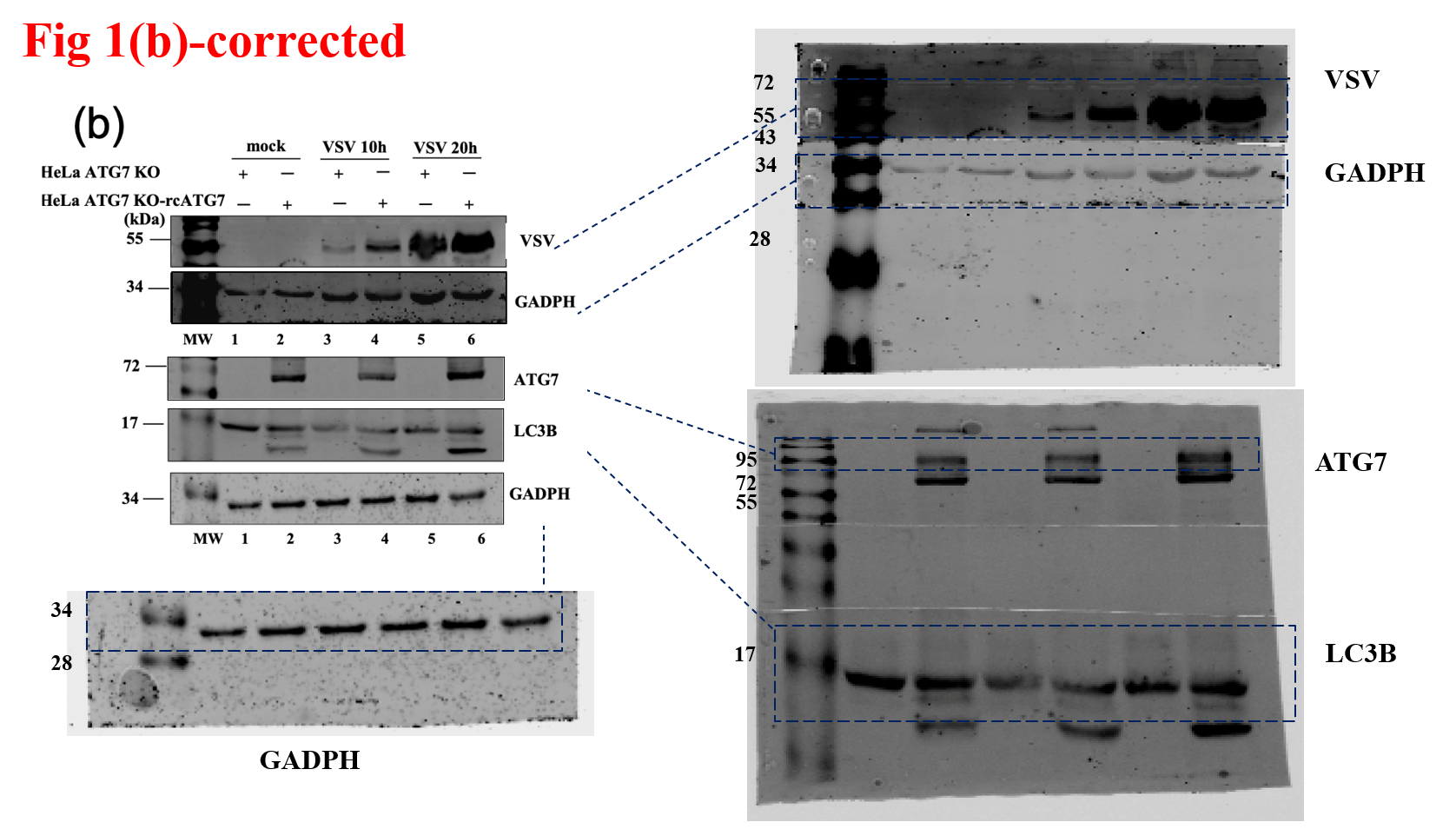

Supplement: Supplementary file 1 [file pathogens-15-00404-s001.zip › Fig1(b).png]

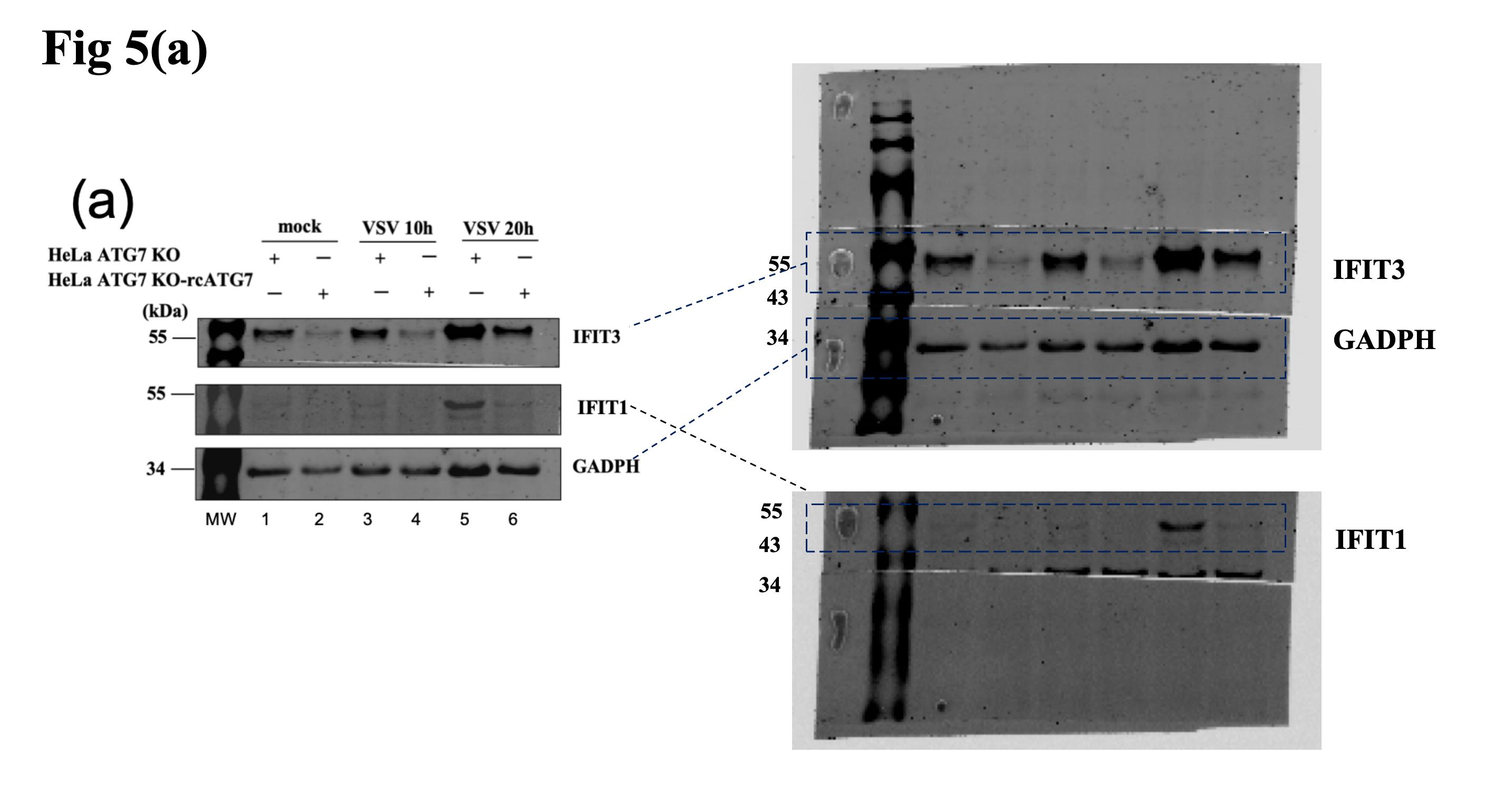

Supplement: Supplementary file 1 [file pathogens-15-00404-s001.zip › Fig5(a).jpg]

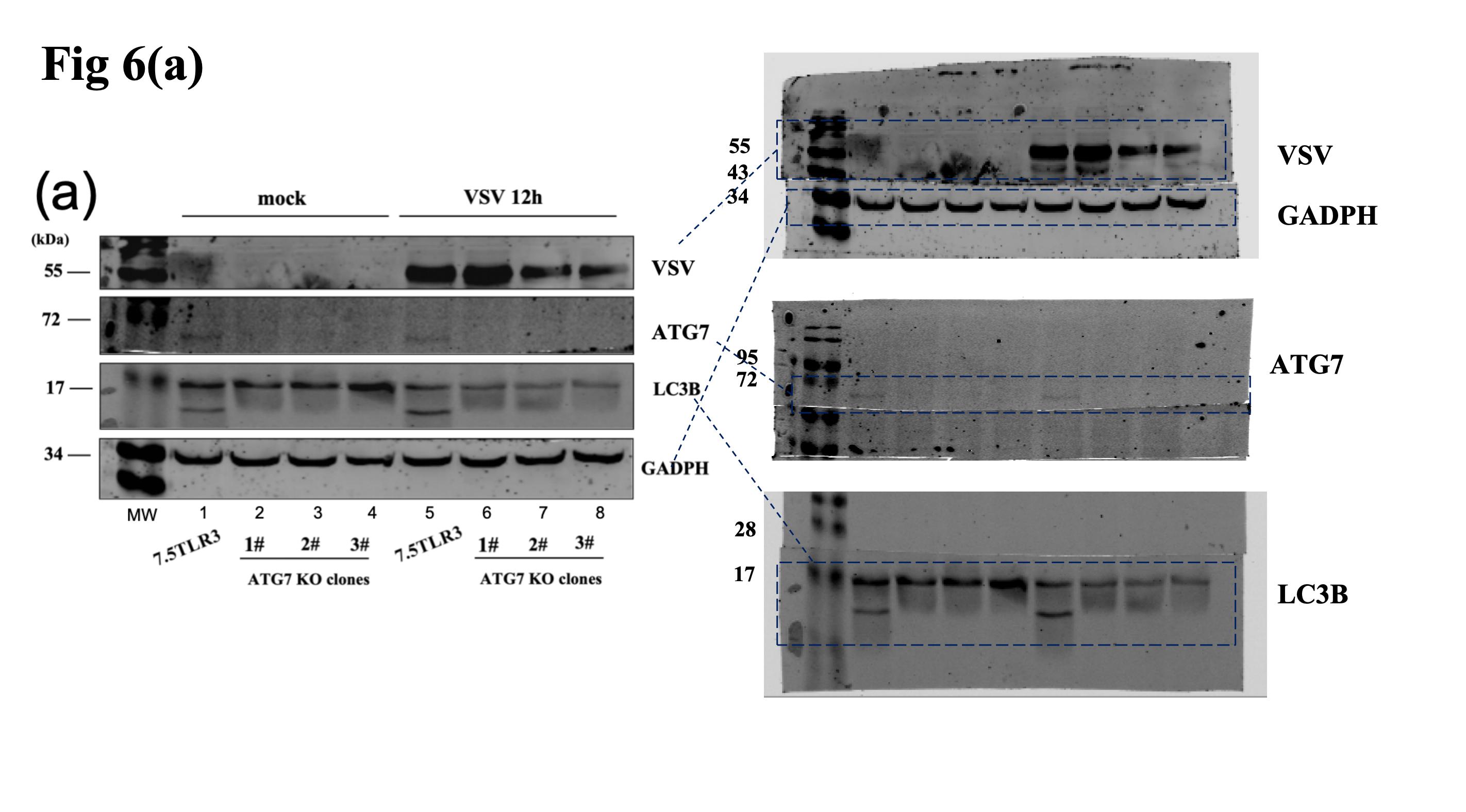

Supplement: Supplementary file 1 [file pathogens-15-00404-s001.zip › Fig6(a).jpg]

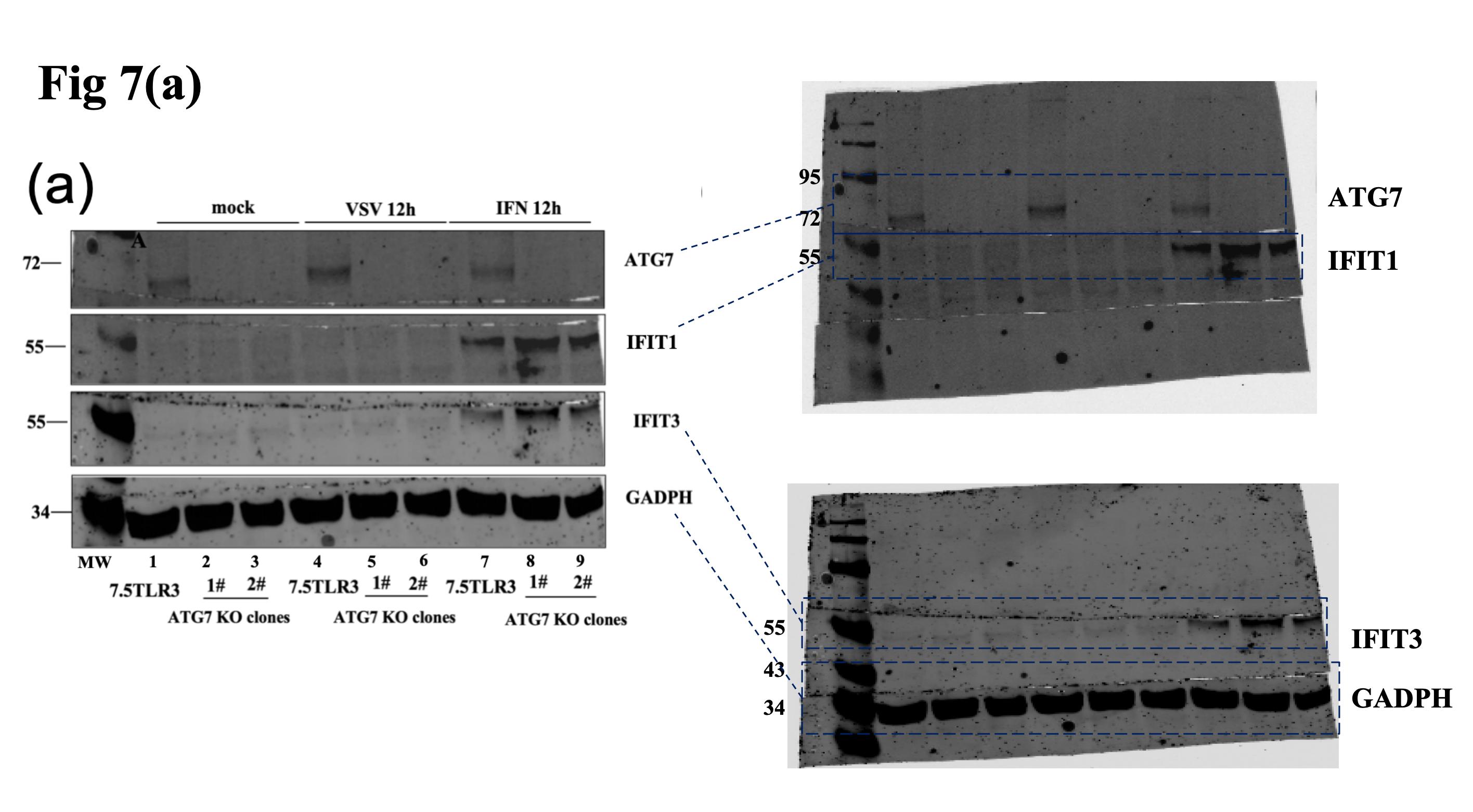

Supplement: Supplementary file 1 [file pathogens-15-00404-s001.zip › Fig7(a).jpg]

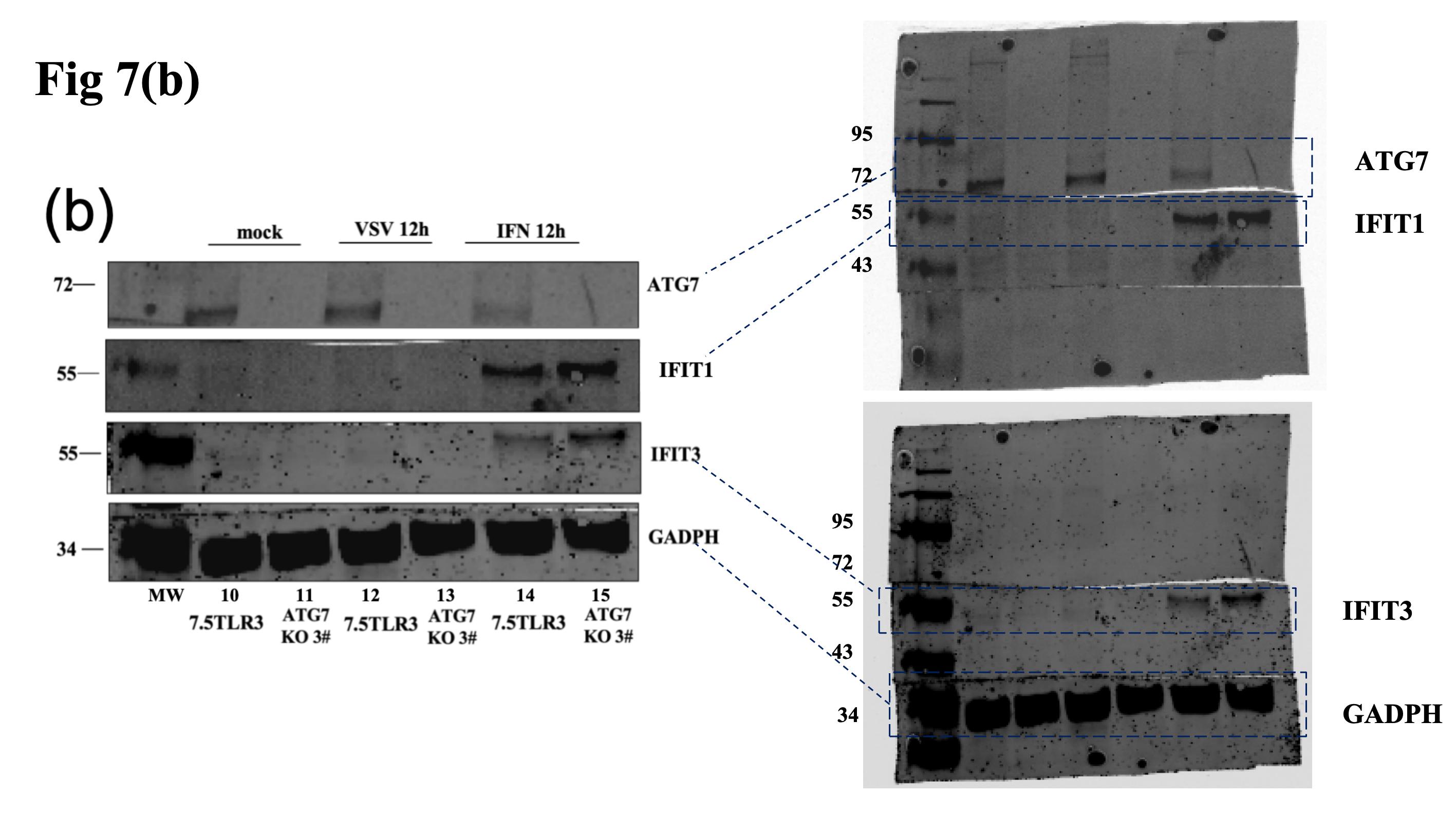

Supplement: Supplementary file 1 [file pathogens-15-00404-s001.zip › Fig7(b).jpg]
